# Effect of Drying Preconditioning and Particle Size on Extractable Constituents from *Plectranthus amboinicus* Leaves by Ultrasound-Assisted Extraction

**DOI:** 10.3390/foods15213795

**Published:** 2026-10-23

**Authors:** Dayana Portillo-Izquierdo, Diego C. Durán, Oswaldo Osorio-Mora, Ramiro Torres-Gallo, Diego F. Tirado

**Affiliations:** 1 Biological Engineering Program, Dirección Académica, Universidad Nacional de Colombia, Sede de La Paz, La Paz 202017, Colombia; dportilloi@unal.edu.co (D.P.-I.); diduran@unal.edu.co (D.C.D.); 2 Grupo de Investigación GAIDA, Facultad de Ingeniería Agroindustrial, Universidad de Nariño, Pasto 520002, Colombia; osorio_oswaldo@udenar.edu.co; 3 Department of Agroindustrial Engineering, Facultad de Ingeniería, Universidad del Atlántico, Puerto Colombia 081001, Colombia; ramirotorres@mail.uniatlantico.edu.co

**Keywords:** drying kinetics, Midilli–Küçük model, effective moisture diffusivity, thymol, carvacrol, thymol-equivalent response, response surface methodology

## Abstract

Thymol and carvacrol are bioactive monoterpenoids of recognized pharmacological value, and *Plectranthus amboinicus* leaves are a promising natural source for their recovery through ultrasound-assisted extraction. This study evaluated how forced-convection drying temperature (36–64 °C) and particle size affect dehydration kinetics and the recovery of thymol and carvacrol (thymol-equivalent response), total phenolic compounds (TPC), and extraction yield. The Midilli–Küçük model best fitted the drying kinetics (R^2^ > 0.99), with effective moisture diffusivity ranging from 6.68 × 10^−12^ m^2^ s^−1^ to 1.72 × 10^−11^ m^2^ s^−1^ and an activation energy of 41.19 kJ mol^−1^. Response surface methodology using a central composite design indicated that particle size had the strongest influence on the evaluated extraction responses. Multi-response optimization identified 46 °C and 0.18 mm as a model-predicted candidate condition, for which the fitted cubic models predicted 785.3 ppm thymol equivalents, 206.4 mg GAE g^−1^ dry extract for TPC, and 10.1% extraction yield. Experimental verification yielded relative prediction errors of −18.6%, +5.6%, and −34.4% for the thymol-equivalent response, TPC, and extraction yield, respectively. These discrepancies, particularly for the thymol-equivalent response and extraction yield, indicate limitations in predictive accuracy. Furthermore, the UV–Vis assay did not establish the specific identity of thymol or carvacrol in the extracts. The findings provide insight into the effects of drying preconditioning and particle size on the measured extraction responses under the evaluated conditions, while further chromatographic analysis and experimental verification are needed to establish compound-specific recovery and the robustness of the predicted conditions.

## 1. Introduction

Oregano (*Plectranthus amboinicus* Lour.) is an herbaceous, aromatic, and perennial plant belonging to the Lamiaceae family, commonly known as French oregano, Mexican mint, Indian oregano, or broadleaf thyme [1]. Oregano is native to the African continent, with a natural range that includes regions of eastern and southern Africa, the Arabian Peninsula, and India; however, it has now been introduced and is widely cultivated in tropical and subtropical regions around the world, especially in dry areas [2]. This species, characterized by a creeping or climbing habit, has traditionally been used for medicinal purposes, as animal feed, and in food and environmental applications [3].

Several studies have documented its use in traditional medicine for the treatment of respiratory, gastrointestinal, and dermatological conditions [4], which is associated with its complex phytochemical composition. In this context, the presence of bioactive compounds such as polyphenols, flavonoids, phenolic acids, terpenoids, and essential oils has been reported, with metabolites such as thymol and carvacrol standing out [4,5]. These compounds have been linked to antimicrobial [6], antioxidant [7], anti-inflammatory [6], insecticidal [8], and anticancer [9] activities, among other pharmacological properties described in the scientific literature [10].

From an anatomical standpoint, the biosynthesis and accumulation of essential oils occur mainly in glandular trichomes present on the leaf surface. In *P. amboinicus*, capitate trichomes, including short- and long-stalked types, specialized in the secretion and storage of these compounds have been described; these compounds accumulate in subcuticular cavities formed by secretory cells [11]. These structures produce complex mixtures of monoterpenes, in which carvacrol and thymol predominate, together with other compounds such as γ-terpinene, *p*-cymene, and thymohydroquinone, which have been reported in the chemical profile of the essential oil of this species [5,12]. The anatomical organization of these secretory structures, documented by optical microscopy (see Section 2.3), comprises glandular trichomes associated with a differentiated mesophyll. The production of these metabolites can be modulated by environmental factors such as radiation and temperature, which influence the regulation of essential oil biosynthesis in these secretory structures [11].

The efficient recovery of bioactive metabolites from plant matrices largely depends on the processing conditions applied to the plant material. In this context, extraction constitutes a decisive step for obtaining essential oils and other compounds of interest [13]. Prior conditioning by drying is particularly relevant to ensure biomass stability, since reducing moisture content and water activity helps limit microbial proliferation and preserve the integrity of the metabolites present [14]. This process involves simultaneous heat and mass-transfer phenomena and can be carried out using different operating configurations, including natural or forced-convection systems [15].

In recent years, the development of more sustainable extraction technologies has promoted the implementation of alternative methods for recovering bioactive compounds from plant matrices [16]. Among these, ultrasound-assisted extraction has been widely studied for its ability to induce acoustic cavitation phenomena, which favor the disruption of cellular structures and improve mass transfer between the plant matrix and the solvent. As a result, this method can increase the recovery yield of metabolites and reduce processing time and operating costs compared with conventional techniques [17]. Likewise, several studies have shown that both drying conditions [14,18] and the extraction method [13,16,17] significantly influence process efficiency and the chemical composition of the compounds obtained from aromatic plant matrices.

Previous research [17] has optimized ultrasound-assisted extraction of thymol from *Plectranthus amboinicus* by evaluating extraction parameters such as temperature, time, and solid-to-solvent ratio. Building on this work, the present study focused on two complementary pre-extraction variables that have not been addressed jointly in the same optimization framework: convective drying temperature and particle size. Rather than re-optimizing the ultrasound extraction conditions, the study investigated how thermal preconditioning and particle-size reduction influence drying kinetics and the subsequent recovery responses under fixed ultrasound-assisted extraction conditions. This approach provides additional insight into the role of raw-material preparation in the extraction process and evaluates whether these preconditioning variables can improve the recovery of extractable constituents. In this context, the present study aimed to model the drying kinetics of *P. amboinicus* leaves, to determine the moisture transfer parameters associated with the process, and to optimize the drying and particle-size conditions to maximize the recovery of thymol and carvacrol (thymol-equivalent response), total phenolic compounds (TPC), and extraction yield by ultrasound-assisted extraction.

## 2. Materials and Methods

### 2.1. Raw Material

Oregano (*Plectranthus amboinicus* Lour.) leaves used in the central composite design (CCD; see Section 2.10) experiments were collected between February and April from the same plant population at the Universidad Nacional de Colombia, Campus de La Paz (10.3889° N, 73.1984° W). Leaves were selected based on morphological criteria, including color, size, and the absence of visible damage. Lower leaves were harvested, washed to remove surface impurities, and processed on the same day of each drying stage. The leaf petioles were removed, and the laminae were cut into sections approximately 1.5 cm long and 1.0 cm wide before convective drying. The drying stages were performed sequentially at 50 °C, 40 °C, 60 °C, 36 °C, and 64 °C. After each stage, the dried material was milled for 10 s using a POLYMIX System PX-MFC 90D mill (KINEMATICA AG, Malters, Switzerland) and sieved to obtain the particle-size fractions required for the 13 CCD experimental runs. Thus, the different particle-size treatments at each drying temperature were obtained from material processed during the corresponding drying stage rather than from separate harvests. Once all required particle-size fractions had been prepared, ultrasound-assisted extractions were performed in the randomized order generated by Design-Expert^®^ software version 13 (Stat-Ease Inc., Minneapolis, MN, USA).

Experimental verification was performed using a separate group of leaves harvested in May from younger plants propagated from the original plant population and grown in another area of the same university. This material was used exclusively for experimental verification and was not included in the CCD experiments. Because the drying stages were performed sequentially using plant material collected over several months, potential biological variability associated with the harvesting schedule cannot be completely excluded when interpreting the effects of drying temperature.

### 2.2. Reagents

This study used absolute ethanol (HPLC, UV, and IR grade, PanReac, Castellar del Vallès, Spain), thymol (certified reference standard, ≥99.9%, GC, Sigma-Aldrich, Taufkirchen, Germany), carvacrol (certified reference standard, ≥99.9%, GC, Sigma-Aldrich, Germany), Folin & Ciocalteu reagent (2 M with respect to acid, Sigma-Aldrich, Germany), sodium carbonate (Na_2_CO_3_, 99.5%, PanReac AppliChem-ITW Reagents, PanReac, Spain), gallic acid (99%, Alfa Aesar, Ward Hill, MA, USA), and methylene blue (1% *w*/*v* aqueous solution, Loba Chemie, Mumbai, India). All reagents were used as received.

### 2.3. Preparation and Photography of the Leaf-Blade Cross-Section

Cross-sections of the leaf blade of *P. amboinicus* were obtained by manual sectioning with a razor blade, using a piece of carrot as supporting tissue [19]. The sections were collected in water, and the thinnest ones were selected. The sections were stained with methylene blue to increase contrast between the mesophyll structures and the glandular trichomes [20], mounted in water on glass slides, and examined under an optical microscope (Primo Star, Carl Zeiss, Suzhou, China) using a 40× objective lens. Photographic records were obtained using a high-resolution smartphone camera coupled to the microscope eyepiece. Representative micrographs are presented in Figure 1.

Figure 1 provides anatomical context for the study by illustrating the leaf tissues and glandular trichomes associated with essential oil storage. It is included to contextualize the plant structures relevant to compound recovery, rather than as evidence of trichome disruption or a direct explanation of the extraction results.

### 2.4. Drying

Samples of *P. amboinicus* were subjected to drying in a forced-convection oven (BINDER GmbH, model FED 115, Tuttlingen, Germany), at the temperature corresponding to each experimental run (see Section 2.10), with 100% fan operation and measured chamber relative humidity of 52–60%. Leaf sections were arranged in a single layer without overlap on trays positioned parallel to the airflow, at an estimated loading of approximately 0.48 kg m^−2^. Air velocity was not measured directly. The manufacturer specifies 32 air changes h^−1^ at 100 °C in an unloaded chamber with the fan set to 100%. The same model has been reported to achieve an air velocity of 1.5 m s^−1^ at 70 °C [21]. Drying curves at 40 °C, 50 °C, and 60 °C were obtained from three drying replicates. At each sampling time, two mass readings were recorded for each replicate and averaged to obtain the mass value used for the drying curve. The two readings were treated as repeated measurements within each replicate, not as independent experimental replicates. Results are reported as mean ± standard deviation across the three drying replicates. Initial moisture content was determined gravimetrically at 103 °C until constant mass (≤0.1% mass variation), following AOAC [22].

### 2.5. Drying Kinetic Modeling

Experimental drying data for *P. amboinicus* leaves at different temperatures were fitted using five empirical thin-layer kinetic models widely reported in the literature [23] to describe moisture transfer behavior during the drying process: Page [24], Modified Page [25], Henderson and Pabis [26], Logarithmic [27], and Midilli–Küçük [28]. The mathematical formulations of the evaluated models are presented in Table S1.

Nonlinear fitting of the experimental curves and estimation of the kinetic parameters were performed using IBM SPSS Statistics v32 (IBM, Armonk, NY, USA) and MATLAB (R2026b, MathWorks, Natick, MA, USA). The predictive capacity of each model was evaluated using the coefficient of determination (*R*^2^), the root mean square error (RMSE), the sum of squared errors (SSE), and the reduced chi-square (χ2), calculated using Equations (1)–(3), respectively. Models with higher *R*^2^ values and lower RMSE and χ2 values were considered the most suitable for describing the drying kinetics of *P. amboinicus* leaves.

(1) $$R^{2}=1-\frac{\sum_{i=1}^{N}{\left(MR_{cal}-MR\right)}^{2}}{\sum_{i=1}^{N}{\left(\bar{MR_{cal\;}}-MR_{exp}\right)}^{2}}$$

(2) $$RMSE={\left[\frac{1}{N}\sum_{i=1}^{N}{\left(MR_{cal}-MR_{exp}\right)}^{2}\right]}^{1/2}$$

(3) $$\chi^{2}=\frac{{\sum_{i=1}^{N}\left(MR_{cal}-MR_{exp}\;\right)}^{2}\;}{N-Z}$$

where *N* is the number of observations; *Z* is the number of model parameters; and *MR_cal_* and *MR_exp_* are the calculated and experimental moisture ratio (*MR)*, respectively.

### 2.6. Effective Moisture Diffusivity and Activation Energy

In the models described in Table S1, *MR* represents the dimensionless moisture, which was determined using Equation (4), as follows:

(4) $$MR=\frac{x_{t\;}-\;x_{e}}{x_{0}-{\;x}_{e}}$$

where *x_t_* is the moisture content of the matrix at time (t), *x*_e_ is the equilibrium moisture content of the matrix, and *x*_0_ is the initial moisture content. Because *x*_e_ is considerably smaller than *x*_t_ and *x*_0_ under the forced-convection drying conditions employed [14], *MR* was simplified as shown in Equation (5):

(5) $$MR=\frac{x_{t\;}}{x_{0}}$$

Effective moisture diffusivity (*D_eff_*) was estimated by fitting Fick’s second law of diffusion to the experimental drying data obtained in this study, consistent with the falling-rate period that dominated the observed drying kinetics. The fit assumed a uniform initial moisture distribution, constant effective diffusivity, negligible shrinkage, and infinite-slab geometry. The analytical solution proposed by Crank [29] for this type of system was applied, as expressed in Equation (6).

(6) $$MR=\frac{8}{\pi^{2}}\sum_{i=0}^{\infty }\frac{1}{{\left(\;2\;i+1\;\right)}^{2}}\;exp\;\left(-\frac{{\left(2i+1\right)}^{2}\pi^{2}\;D_{eff}t}{4\;L^{2}}\right)$$

where *D_eff_* corresponds to the effective moisture diffusivity (m^2 ^s^−1^), *L* represents half the thickness of the sample (m), and *t* is the drying time (s). Thickness was measured on 10 fresh leaves at three positions per leaf (basal, middle, and apical), avoiding the central midrib, using a digital caliper with a resolution of 0.01 mm. *L* was calculated as half the mean thickness. For sufficiently long drying times, the general solution was simplified by considering only the first term of the Crank series, giving Equation (7). Subsequently, applying the natural logarithm yielded a linear relationship between *Ln (MR)* and *t*, as shown in Equation (8). The slope of the resulting linear relationship corresponded to the kinetic constant *K*, which is related to *D_eff_* through Equation (9), considering one-dimensional diffusion in slab geometry. Finally, the influence of temperature on *D_eff_* was described using the Arrhenius equation (Equation (10)), allowing the activation energy (*A_e_*) and the pre-exponential factor *D_0_* to be estimated from the linearization of *Ln (D_eff_)* as a function of T^−1^.

(7) $$MR=\frac{8}{\pi^{2}}\;exp\;\left(-\frac{\pi^{2}\;D_{eff}t}{4\;L^{2}}\right)$$

(8) $$Ln\;(MR)=Ln\;\frac{8}{\pi^{2}}-\frac{\pi^{2}\;D_{eff}\;t}{4\;L^{2}}$$

(9) $$K=\frac{\pi^{2}\;D_{eff}}{4{\;L}^{2}}$$

(10) $$D_{eff}=D_{0}\;exp\;\left(-\frac{A_{e}}{RT}\right)$$

where *R* is the universal gas constant (8.314 × 10^−3^ kJ mol^−1^ K^−1^), and *T* is the absolute temperature (K).

### 2.7. Ultrasound-Assisted Extraction

Ultrasound-assisted extraction was carried out using ethanol as the extraction solvent. The dried sample (140 mg) was mixed with 5.0 mL of ethanol, corresponding to a solid-to-solvent ratio of 1:35 *w*/*v*, and extracted in an Elmasonic Easy 30 H ultrasonic bath (Elma Schmidbauer GmbH, Singen, Germany; 37 kHz, 280 W total power, 80 W rated ultrasonic power) at 55 °C for 23 min. Sample temperature was continuously monitored using a thermocouple connected to a multimeter and immersed in the extraction vessel; ice was added to the surrounding bath as needed to keep the sample at 55 °C. Subsequently, the samples were centrifuged at 1449× *g* for 5 min, and the supernatant was used for UV-Vis analysis (Agilent Cary 60 UV-Vis, Agilent Technologies, Santa Clara, CA, USA). The extraction conditions were selected based on previously reported ultrasound-assisted extraction conditions for thymol from *P. amboinicus* [11,15]. The ultrasound-assisted extraction setup can be seen in Figure 2.

### 2.8. Thymol-Equivalent Response

The thymol-equivalent response was determined by UV–Vis spectrophotometry at 275 nm using an external calibration curve prepared with a certified thymol reference standard. The wavelength was selected based on the reported maximum ultraviolet absorption of thymol in ethanol (274 nm–276 nm) and previous applications of direct UV–Vis determination [30,31]. An independent calibration curve was also prepared with a certified carvacrol reference standard at 273 nm, under the same solvent and instrumental conditions, to evaluate the potential overlap of the two phenolic monoterpenes.

Thymol and carvacrol calibration curves are provided in Figure S1. The slopes differed by approximately 0.85%, and their difference was not statistically significant (*p* = 0.757), indicating very similar UV responses in this spectral region. These results show that absorbance at 275 nm cannot by itself provide unequivocal selectivity for thymol in the crude leaf extract. Accordingly, the measured response is reported as a thymol-equivalent UV–Vis response based on the external thymol standard, rather than as chromatographically confirmed thymol. Before measurement, extracts were diluted tenfold with absolute ethanol. The calibration-derived concentration therefore refers to the diluted analytical solution; the original-extract concentration was obtained by applying the 10-fold dilution factor. For comparison among treatments, the corrected concentration was also expressed as mg thymol equivalents (TE) per gram of dry leaves using the 5.0 mL extraction volume and 0.140 g dry-sample mass.

### 2.9. Determination of Total Phenolic Content and Extraction Yield

Extraction yield (% *w*/*w*) was determined gravimetrically by evaporating a known aliquot of the extract in a water bath at 60 °C. The dry residue obtained from the aliquot was used to estimate the total dry extract mass, accounting for the total extract volume. Extraction yield was calculated as the ratio of the estimated total dry extract mass to the initial dry leaf mass, multiplied by 100%. The dry extract was then reconstituted in absolute ethanol at a concentration of 1 mg mL^−1^ and used to determine total phenolic content (TPC) by the Folin–Ciocalteu method. Briefly, 50 µL of extract was mixed with 425 µL of type 1 water and 125 µL of Folin–Ciocalteu reagent. After 6 min, 400 µL of 7.1% *w*/*v* sodium carbonate was added. The mixture was incubated in the dark for 1 h, and absorbance was measured at 760 nm by UV–Vis spectrophotometry. Quantification was performed using a calibration curve prepared with gallic acid as the standard, and results were expressed as milligrams of gallic acid equivalents per gram of dry extract (mg GAE g^−1^ dry extract).

### 2.10. Experimental Design and Statistical Analysis

To identify model-predicted extraction conditions for *P. amboinicus*, an RSM was used. A CCD was employed to evaluate the independent variables and their interactions. The experimental data were initially evaluated using a second-order polynomial model, in accordance with the conventional approach employed for central composite designs. Model adequacy was assessed based on analysis of variance (ANOVA), the lack-of-fit test, and model fit statistics. When the quadratic model exhibited a significant lack of fit (*p* ≤ 0.05), higher-order polynomial terms were subsequently evaluated to determine whether the lack of fit could be adequately explained by additional model terms. Statistically significant cubic terms were identified for the corresponding response(s), and a reduced cubic model was therefore fitted by retaining only the significant cubic term(s) and the relevant lower-order terms required by model hierarchy. A reduced cubic model was selected when the inclusion of a third-order term resulted in a statistically significant improvement in model adequacy and yielded a non-significant lack of fit (*p* > 0.05). The adequacy of the selected reduced cubic model was further confirmed by the absence of significant lack of fit and by acceptable agreement between the adjusted and predicted coefficients of determination. Model selection was further supported by the adjusted and predicted coefficients of determination (*R^2^*_adj and *R^2^*_pred, respectively), the coefficient of variation, and adequate precision.

Simultaneous optimization of the three responses was carried out with a reduced number of experiments, using drying temperature and particle size as independent variables owing to their significant reported influence on the extraction efficiency of compounds from biological matrices. Each factor was evaluated at five coded levels, from the lower axial point (−α) to the upper axial point (+α), with α = 1.414: drying temperature ranged from 36 °C to 64 °C, with 40 °C, 50 °C, and 60 °C as factorial levels and 50 °C as the center point; particle size ranged from 0.19 mm to 2.31 mm, with 0.50 mm, 1.25 mm, and 2.00 mm as factorial levels and 1.25 mm as the center point. The complete coded and actual levels of each independent variable are presented in Table S2.

The rotatable CCD comprised a total of 13 experimental runs, consisting of four factorial points, four axial points, and five center points (see Table S2). The runs were performed in randomized order, and each condition was evaluated in triplicate, resulting in a total of 39 experimental trials; these replicates did not represent independent biological batches. Extraction yield (%), thymol-equivalent response (ppm), and TPC (mg GAE g^−1^) were selected as response variables to evaluate the individual and combined effects of drying temperature and particle size. The experimental design was developed using Design-Expert^®^. The interaction between factors and the significance of the effects were evaluated by ANOVA, considering a significance level of 95% (*p* ≤ 0.05). The predictive capacity of the model was determined through the coefficient of determination (*R^2^*), along with the adjusted and predicted coefficients of determination (R^2^_adj and R^2^_pred, respectively), to assess model fit and predictive performance.

## 3. Results and Discussions

### 3.1. Drying Curves

The initial moisture content of the *P. amboinicus* leaves was 95 ± 1% (wet basis, *wb*). As shown in Figure 3, the moisture content decreased continuously during convective drying at all temperatures, showing a marked influence of temperature on the dehydration kinetics. The time required to reach the equilibrium moisture content decreased progressively with increasing temperature, from approximately 1800 min at 40 °C, to 1140 min at 50 °C, and to 960 min at 60 °C, confirming that increasing temperature accelerates the drying kinetics. Similar results have been reported for aromatic and medicinal leaves dried by convection (i.e., tea), where temperature was one of the operating variables with the greatest influence on the overall rate of moisture removal [32]. Meanwhile, a previous study reported that leaves of *Origanum vulgare* dried at 50 °C required 24 h to reach a residual moisture content of 5.3%, whereas a shorter drying time was observed in the present study under the same temperature [18]. This discrepancy may be related to differences in plant tissue structure, sample thickness, and drying operating conditions.

The curves obtained showed the characteristic behavior of biological materials whose dehydration occurs predominantly during the falling-rate period. In this stage, the drying rate decreases progressively as the material’s water content is reduced, a behavior widely reported for aromatic and medicinal leaves subjected to convective drying [33]. The effect of temperature on the observed kinetics was reflected in the reduction in the time needed to reach equilibrium moisture content, showing an improvement in the overall process efficiency [34].

### 3.2. Effective Diffusion Coefficient and Activation Energy

*D_eff_* was estimated from the analytical solution of Fick’s second law for a flat slab, assuming that internal moisture transfer is the main resistance during the falling-rate period. Meanwhile, the characteristic diffusion length (*L*) was defined as one-half of the mean leaf-lamina thickness (*L* = 0.25 mm; see Section 2.6). *D_eff_* increased progressively with temperature, from 6.68 × 10^−12^ m^2^ s^−1^ at 40 °C to 1.35 × 10^−11^ m^2^ s^−1^ at 50 °C and 1.72 × 10^−11^ m^2^ s^−1^ at 60 °C (see Table S3). This increase reflected higher vapor pressure, lower water viscosity, and greater mobility of the water retained in the plant matrix, along with heat-induced microstructural changes favoring moisture migration toward the surface [35]. These results were consistent with the higher drying rates observed experimentally (see Figure 4) and with similar temperature-dependent trends reported for other hot-air-dried aromatic leaves [36].

*D_eff_* values obtained in this study were lower than those previously reported for *P. amboinicus*. Castillo-Téllez et al. [37] reported 1.14 × 10^−10^ m^2^ s^−1^, 2.28 × 10^−10^ m^2^ s^−1^, and 1.03 × 10^−9^ m^2^ s^−1^ for electric-oven drying at 45 °C, 55 °C, and 65 °C, respectively, while Rout et al. [38] found markedly higher diffusivities under microwave drying than under hot-air or vacuum drying. These differences likely reflected the combined effect of temperature, drying method, and the sample geometry and characteristic length used to estimate *D_eff_*.

The substantial reduction in leaf thickness observed after drying (~60%) should be considered when interpreting the estimated effective diffusivities. Since the model used the initial mean leaf thickness and did not account for dimensional changes during drying, the reported *D_eff_* values are apparent estimates that may be affected by shrinkage.

The temperature dependence of *D_eff_* was described by the Arrhenius equation, yielding an *A_e_* of 41.19 kJ mol^−1^ and a pre-exponential factor (*D_0_*) of 5.31 × 10^−5^ m^2^ s^−1^. *A_e_* reflects the thermal sensitivity of internal moisture transport and the minimum energy required to initiate diffusion within the plant matrix [39]. This value was within the range reported for aromatic leaves and other plant materials under convective drying and is close to the 39.30 kJ mol^−1^ reported for purple basil (*Ocimum basilicum* L.) [40], indicating similar temperature sensitivity of moisture diffusion in both matrices. The high coefficient of determination (*R^2^* = 0.93) confirmed that the Arrhenius model adequately described the effect of temperature on *D_eff_* in *P. amboinicus* leaves.

Figure 4 shows the drying rate as a function of time and moisture content at the three temperatures evaluated. All curves displayed an initial high-rate stage followed by a pronounced decline toward near-zero values, reflecting the approach to hygroscopic equilibrium. The nonlinear relationship between drying rate and moisture content, with sharper variations at intermediate moisture levels, suggested progressive changes in the moisture accessible within the tissue as drying proceeds, confirming the non-stationary nature of leaf drying, governed by the interaction between material state and applied thermal conditions.

### 3.3. Drying Kinetic Models

The fitting results revealed that the models analyzed adequately described the drying kinetics of *P. amboinicus*, as shown by the high coefficients of determination (*R^2^* > 0.98) and the low RMSE, SSE, and χ^2^ values. However, the Midilli–Küçük model provided the best statistical fit at all operating temperatures, showing the smallest deviation between experimental and predicted values (see Table 1). This behavior suggested that the mathematical structure of the Midilli–Küçük model more accurately captured the combined mass transfer mechanisms governing the decrease in moisture content during drying.

The predominance of the Midilli–Küçük model observed in the present study agreed with results reported for various plant materials, where this model has shown a high predictive capacity to describe the evolution of the moisture ratio during thin-layer drying. Previous studies identified the Midilli–Küçük model as providing the best fit for the drying kinetics of clove leaves and *Mentha haplocalyx* [39,41]. Likewise, the kinetic parameter *k* varied among the evaluated drying temperatures, consistent with the temperature-dependent behavior reported for drying kinetics in plant materials [42].

Figure 5 shows the evolution of the experimental and estimated *MR* during convective drying of *P. amboinicus* leaves at 40 °C, 50 °C, and 60 °C. In all cases, a continuous decrease in *MR* with time was observed, reaching values close to equilibrium in less time as the drying temperature increased. The closeness between the experimental and calculated values shows the ability of the Midilli–Küçük model to adequately represent the drying kinetics under the conditions evaluated. Similar behavior was reported during the drying of clove leaves, where the Midilli–Küçük model showed the best statistical performance for describing the evolution of the moisture ratio compared with other thin-layer models [43].

### 3.4. Response Surface Optimization

The central composite design and the experimental results of the response variables are presented in Table 2. The UV–Vis thymol-equivalent response ranged from 244.6 ppm to 717.3 ppm in the original extract, corresponding to (8.7–25.6) mg TE g^−1^ dry leaves. TPC ranged from 62.8 mg GAE g^−1^ to 193.4 mg GAE g^−1^, and extraction yield ranged from 2.0% to 9.0%. The highest experimental values for the three responses were obtained at 0.19 mm and 50 °C, whereas the lowest values were observed at 2 mm and 60 °C. These observations indicate a marked influence of particle size and drying temperature on the responses evaluated, but the highest observed response combination should be distinguished from the model-derived multi-response compromise described below.

A cubic polynomial model adequately fitted the experimental data, with low standard errors and *R*^2^ of 0.99 for the thymol-equivalent response, TPC, and yield. The ANOVA of the response surfaces identified that the linear effects of particle size were highly significant for all three responses (*p* < 0.0001), while the linear effect of temperature was significant for the thymol-equivalent response and total phenols, but not for extraction yield. The quadratic terms (A^2^ and B^2^) were significant in all three models, indicating nonlinear behavior of the responses within the experimental domain. Model adequacy was further supported by low coefficients of variation, high adjusted *R*^2^ values, and adequate precision ratios, confirming the reliability of the fitted models for navigating the design space.

Comparison of the initial quadratic models with the reduced cubic models showed that the quadratic formulation was insufficient to adequately describe the experimental responses, as indicated by the significant lack of fit. After inclusion of higher-order terms, the final reduced cubic models showed no significant lack of fit at the 5% significance level for the thymol-equivalent response, TPC, or extraction yield. Notably, the AB^2^ term was highly significant for all three responses (*p* = 0.0005, 0.0011, and 0.0003, respectively) and was retained in the final models. In addition, the A^2^B term was significant for extraction yield (*p* = 0.017) and was also retained in that model.

The regression coefficients obtained (see Tables S4–S6) made it possible to quantify the contribution of the factors studied to the responses evaluated. Particle size showed the largest absolute linear coefficients, showing its predominant influence on the thymol-equivalent response, TPC, and extraction yield. The predominant influence of particle size on the evaluated extraction responses may be explained by several complementary mass-transfer mechanisms. Reducing particle size generally increases the exposed solid–liquid interfacial area and shortens the internal diffusion pathways, potentially facilitating solvent access to extractable constituents and reducing internal mass-transfer resistance. In plant matrices, milling may also expose intracellular and subcellular structures that were less accessible in larger particles. These effects can facilitate the release of extractable constituents into the surrounding solvent, although the extent of structural disruption depends on the mechanical properties and anatomical organization of the tissue.

These mechanisms may be particularly relevant during ultrasound-assisted extraction. Acoustic cavitation generates localized mechanical effects and fluid motion that can improve solvent–solid contact and enhance mass transfer at the particle surface. Consequently, the greater accessibility associated with smaller particles may complement ultrasound-induced transport and mechanical effects, contributing to the higher extraction responses observed within the experimental domain. Nevertheless, the present study did not directly measure specific surface area, cellular disruption, solvent penetration, or cavitation-induced damage. Therefore, these mechanisms should be interpreted as plausible explanations for the observed particle-size effect rather than as experimentally confirmed pathways. Figure 1 provides anatomical context by illustrating the leaf mesophyll and glandular trichomes, but it does not directly demonstrate structural changes caused by milling or ultrasound treatment.

The linear temperature term was statistically significant for both the thymol-equivalent response (*p* = 0.0438) and TPC (*p* = 0.0112). Similar results have been observed in species of the Lamiaceae family, where drying differentially modified the chemical composition of the essential oil, significantly reducing the proportion of more volatile precursor monoterpenes, such as *p*-cymene and γ-terpinene, while more stable phenolic compounds, such as carvacrol, tended to remain unchanged or even increase after thermal processing [18]. Likewise, the significance of the interaction and curvature terms showed the presence of synergies and curvature effects within the experimental space, indicating that the system’s response could not be adequately described by purely linear relationships. In the model equations, $x_{1}$ and $x_{2}$ represented the coded factors corresponding to drying temperature and particle size, respectively, in accordance with the levels established in Table S2. Equation (11), Equation (12), and Equation (13) represented the selected models for the thymol-equivalent response (ppm), TPC (mg GAE g^−1^), and extraction yield (%) of *P. amboinicus*, respectively.

(11) $$\begin{array}{c} Thymol-equivalent\;response\\ \;=457.71-22.98x_{1}-113.03x_{2}+23.45x_{1}x_{2}-55.43x_{1}^{2}\\ \;+40.65x_{2}^{2}-85.79{x_{1}x}_{2}^{2} \end{array}$$

(12) $$\begin{array}{l} TPC=139.68-9.75x_{1}-17.58x_{2}-11.25x_{1}x_{2}-27.35\;x_{1}^{2}+14.98x_{2}^{2}\\ \;-22.20{x_{1}x}_{2}^{2} \end{array}$$

(13) $$\begin{array}{c} Yield=4.34-0.0318x_{1}-1.71x_{2}+0.2925x_{1}x_{2}-0.9{7x}_{1}^{2}+1.07x_{2}^{2}\\ \;+0.602x_{1}^{2}x_{2}-1.52{x_{1}x}_{2}^{2} \end{array}$$

Additionally, 2D contour plots and 3D response surfaces were generated from the cubic model equations to evaluate the behavior of the responses within the experimental domain (see Figure 6). The surfaces obtained showed nonlinear behavior, associated with the significance of the quadratic terms and higher-order interactions (A^2^B and AB^2^), making it possible to identify locally favorable regions and trade-off zones among the thymol-equivalent response, TPC, and extraction yield.

A multi-response desirability approach was applied to jointly maximize the thymol-equivalent response, TPC, and extraction yield, using the importance levels and desirability weights specified in Table 3. The model-predicted preconditioning conditions were a drying temperature of 46 °C and a particle size of 0.18 mm, with an overall desirability of 0.78. These conditions represent the solution selected under the specified optimization criteria rather than an experimentally confirmed optimum.

The numerical optimization was allowed to extend beyond the experimental particle-size range of 0.19 mm–2.31 mm. Consequently, the selected particle size of 0.18 mm represents a slight extrapolation below the lower experimental boundary, rather than a rounding difference. The corresponding model predictions should therefore be interpreted with additional caution. Although the selected condition was subsequently evaluated experimentally, this verification does not establish an optimum or demonstrate model reliability outside the experimental domain.

### 3.5. Validation

The predictive performance of the reduced cubic models was evaluated experimentally in triplicate at the selected preconditioning conditions of 46 °C and 0.18 mm particle size (see Table 3). Absolute relative prediction errors calculated using the experimental values as the denominator were 18.6%, 5.6%, and 34.4% for the thymol-equivalent response, TPC, and extraction yield, respectively. The models underestimated the thymol-equivalent response and extraction yield and overestimated TPC. Agreement was closest for TPC, whereas the larger deviations for the other responses indicate limitations in predictive accuracy at the selected condition.

The discrepancies observed for extraction yield and the thymol-equivalent response may partly reflect variability among the leaf batches used for model development and experimental verification. Differences in chemical composition and essential oil content have been reported among production batches of aromatic Lamiaceae species, even under similar agronomic conditions [44]. In this study, leaves were collected in stages between February and May, and experimental verification used an independent batch harvested in May. Differences in plant developmental stage and growing conditions may therefore have contributed to the observed deviations. Previous studies have reported effects of climatic conditions, soil factors, and environmental stress on essential oil content and secondary metabolism [45,46,47,48]. However, these factors were not independently evaluated in the present study, and their contribution cannot be separated from model prediction error.

## 4. Conclusions

Drying temperature affected dehydration kinetics and estimated effective moisture diffusivity in *Plectranthus amboinicus* leaves, whereas particle size had the greatest influence on the measured extraction responses. The Midilli–Küçük model described the drying behavior over the evaluated temperature range, with an activation energy of 41.19 kJ mol^−1^ and estimated effective moisture diffusivities on the order of (10^−12^–10^−11^) m^2^ s^−1^. The extraction experiments quantified a thymol-equivalent UV–Vis response, total phenolic content (TPC), and gravimetric extraction yield under the conditions investigated. Although multi-response optimization identified 46 °C and 0.18 mm as a model-predicted candidate condition, experimental verification revealed discrepancies between predicted and measured responses, particularly for the thymol-equivalent response and extraction yield. Moreover, the UV–Vis method did not establish the specific identity of thymol, and the potential contribution of differences between leaf batches could not be separated from model prediction error. Therefore, the proposed condition should be regarded as a candidate for further investigation rather than as an experimentally established optimum. Further studies incorporating compound-specific chromatographic analysis, independent biological batches, and additional experimental verification are required to assess the robustness of the predicted extraction conditions.

## Figures and Tables

**Figure 1:**
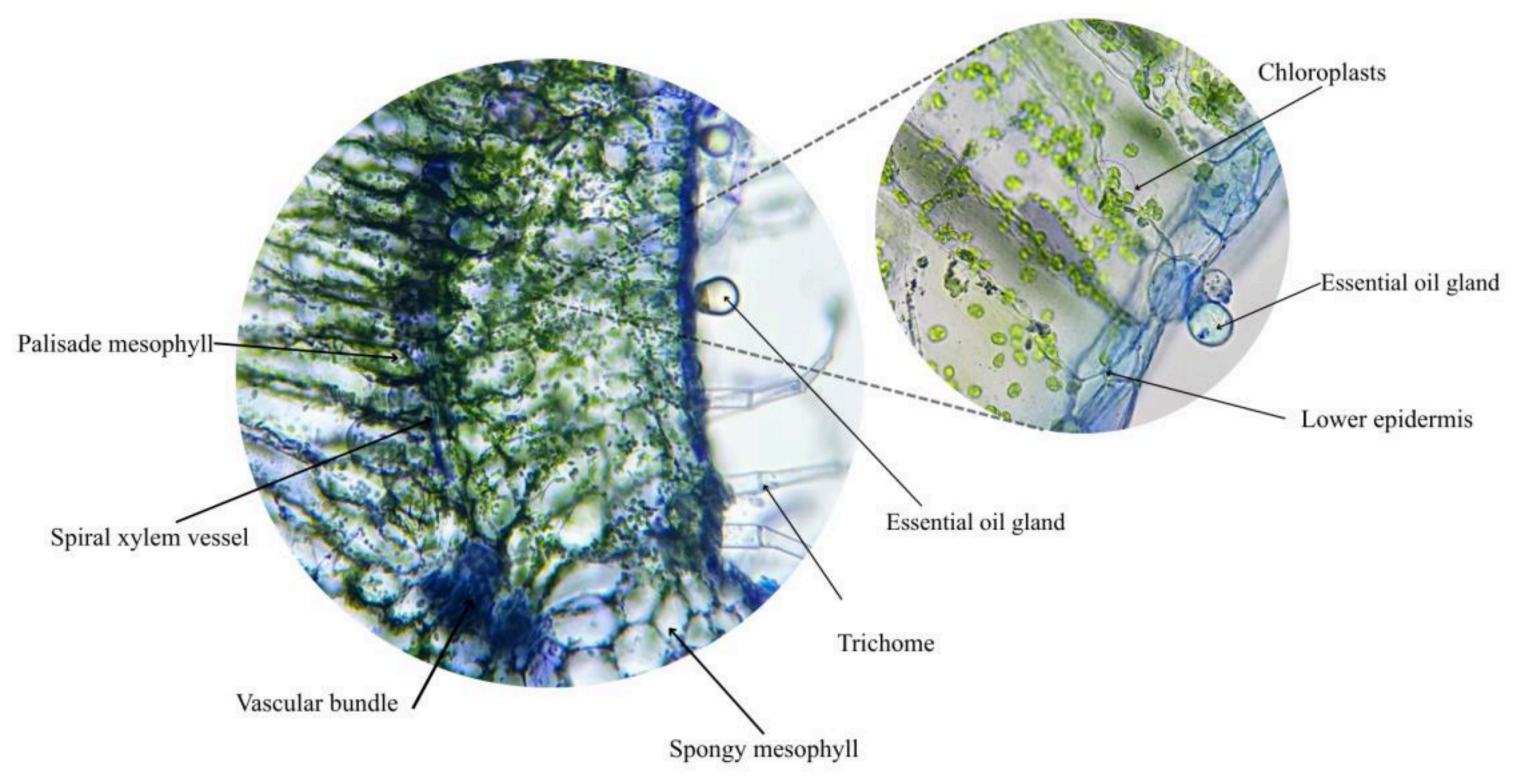
Cross-section of the oregano (*Plectranthus amboinicus*) leaf blade, stained with methylene blue, with glandular trichomes and differentiated mesophyll.

**Figure 2:**
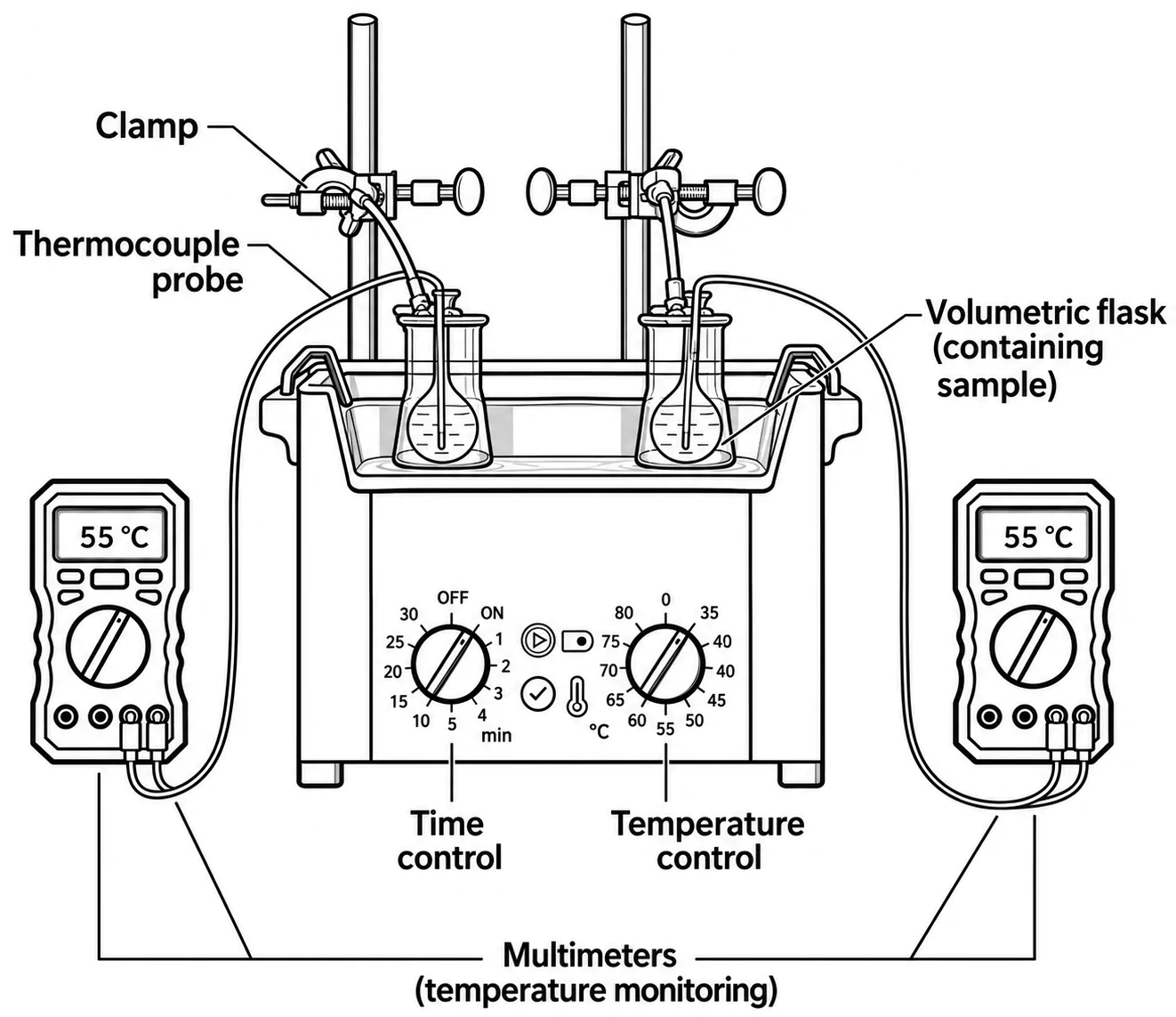
Ultrasound-assisted extraction setup.

**Figure 3:**
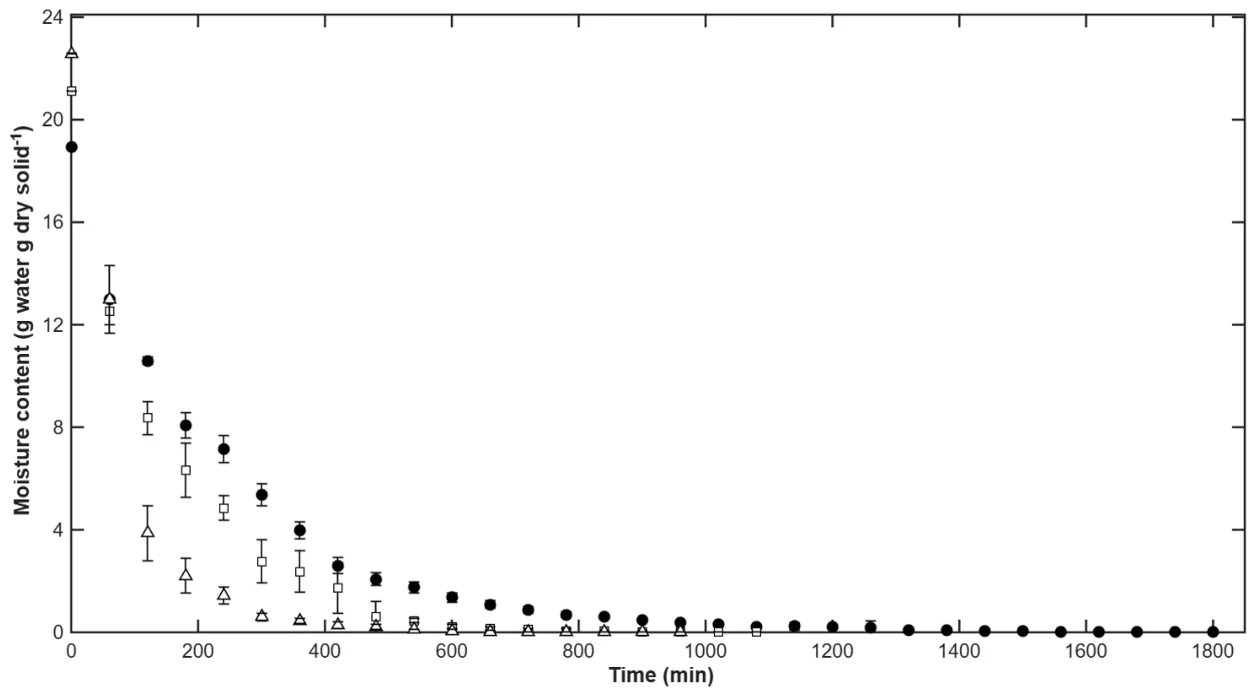
Drying curves of *Plectranthus amboinicus* leaf at 40 °C (●), 50 °C (□), and 60 °C (△).

**Figure 4:**
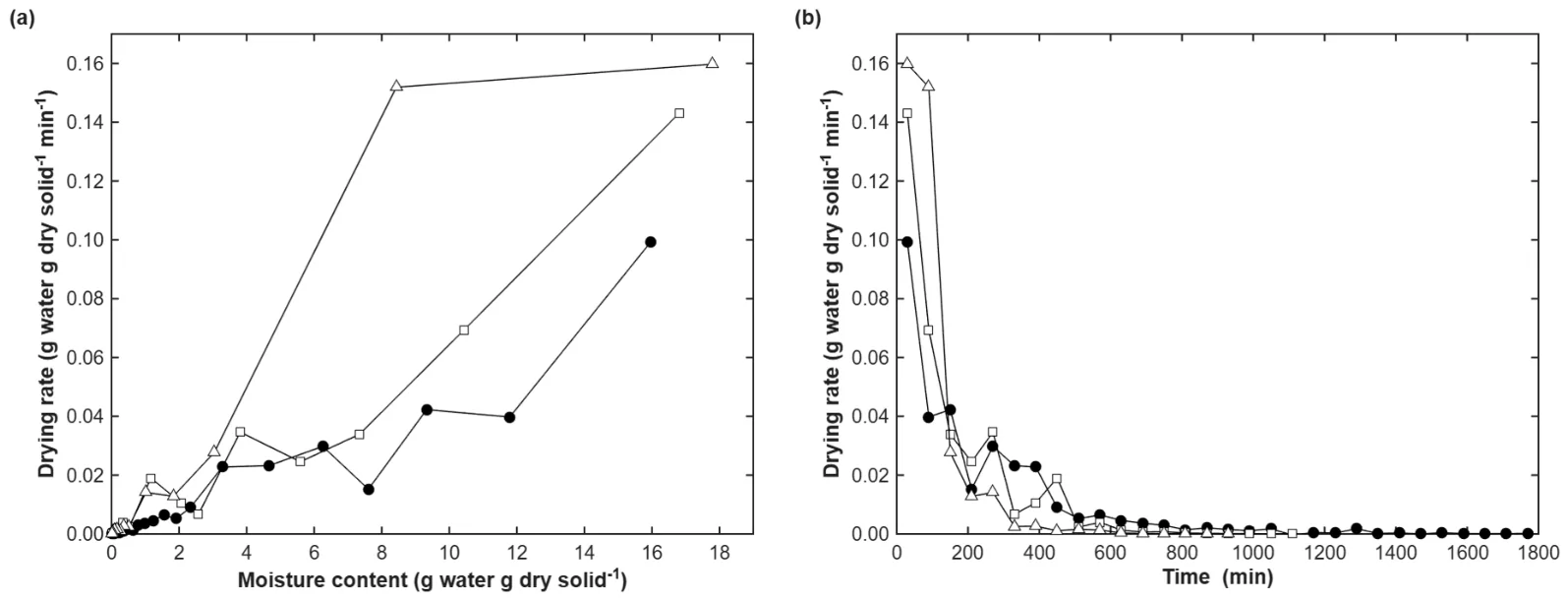
Variation of (**a**) drying rate versus moisture content and (**b**) drying rate versus time for a forced-convection oven at 40 °C (●), 50 °C (□), and 60 °C (∆).

**Figure 5:**
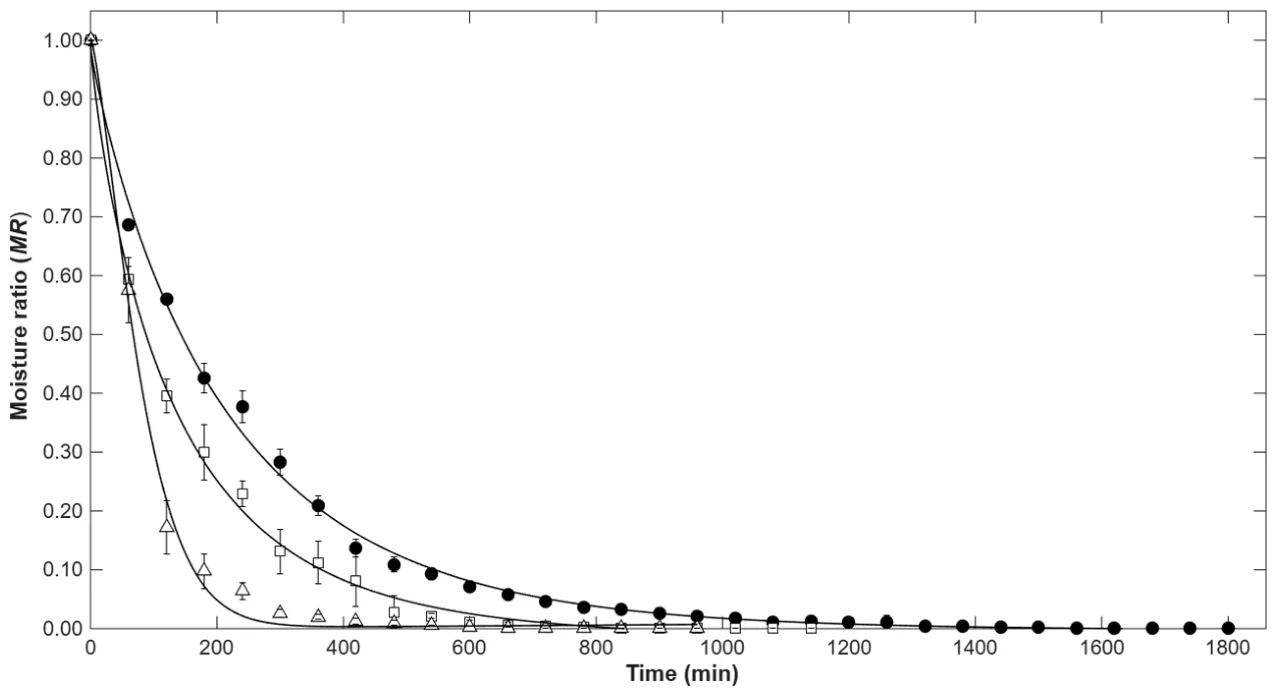
Drying kinetics adjusted by the Midilli–Küçük model of *Plectranthus amboinicus* leaves at 40 °C (●), 50 °C (□), and 60 °C (∆).

**Figure 6:**
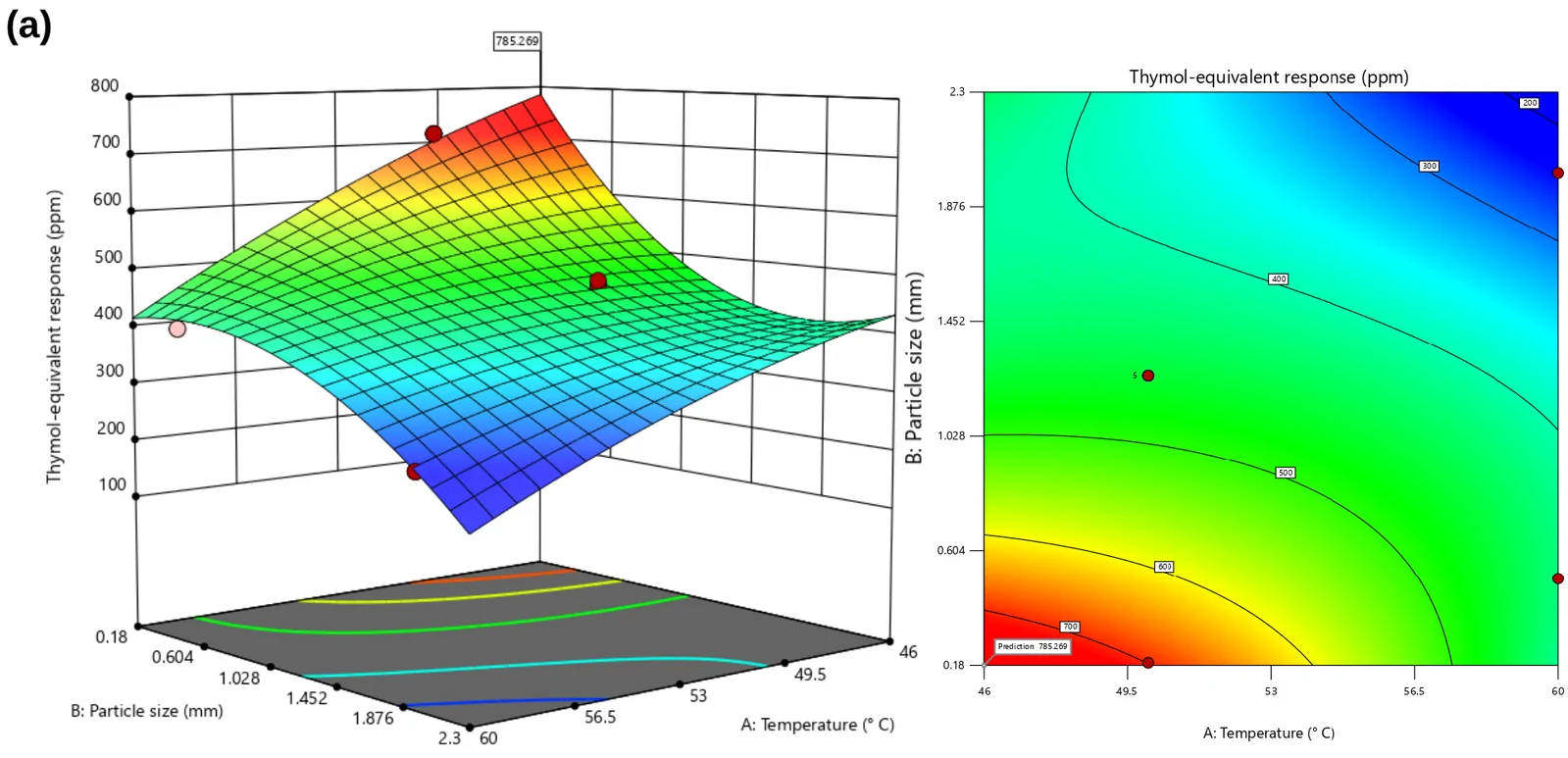

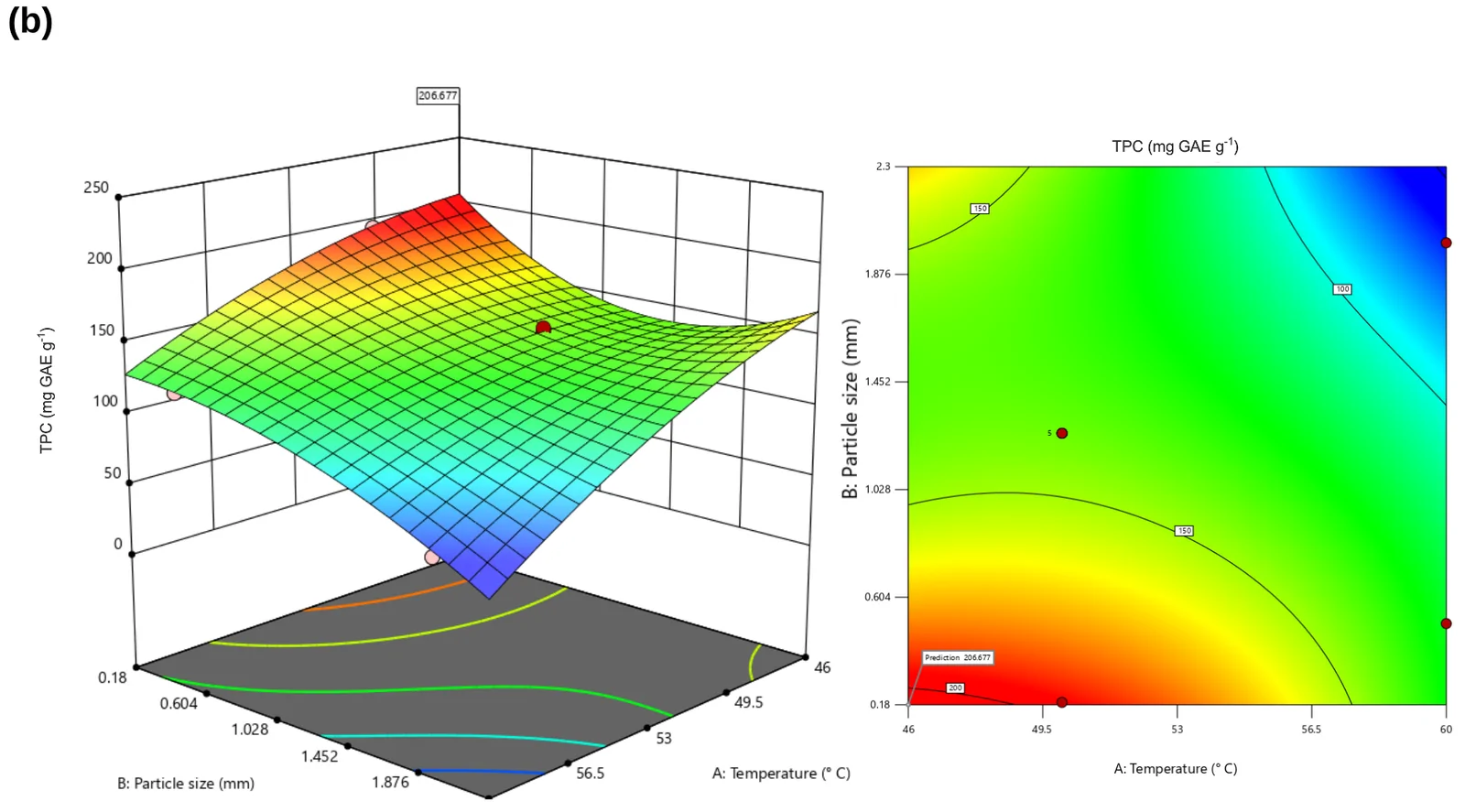

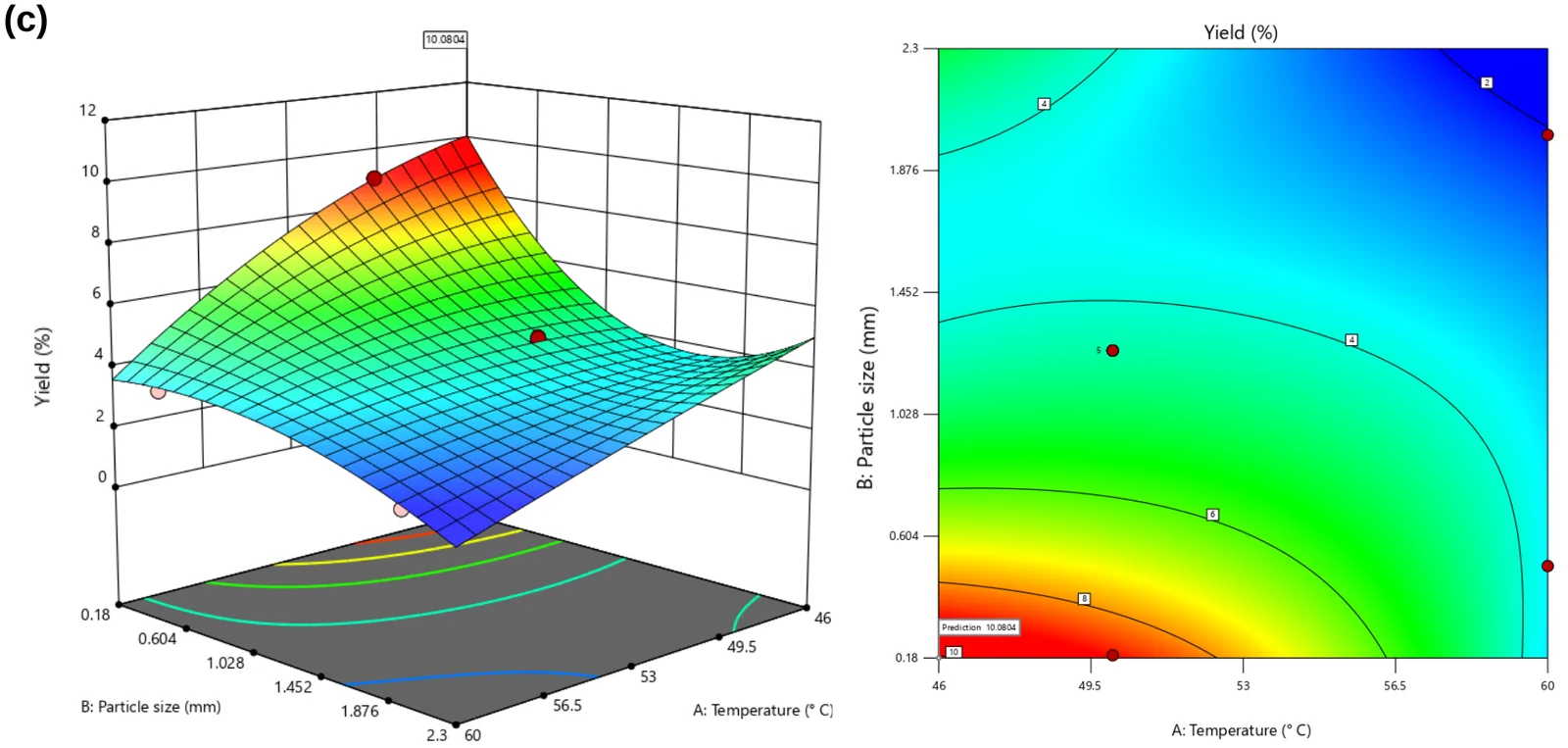
Response surfaces and contour maps obtained by response surface methodology (RSM) for (**a**) thymol-equivalent response (ppm), (**b**) TPC (mg GAE g^−1^ dry extract), and (**c**) yield (% *w*/*w*), as a function of temperature (X_1_) and particle size (X_2_).

**Table 1:** Model parameters and statistical results for the drying of *Plectranthus amboinicus* at 40 °C, 50 °C, and 60 °C.

Model	Parameter	40 °C	50 °C	60 °C
Logarithmic	*k* × 10^−3^	4.4038	6.8274	11.9726
	a	0.9598	0.9650	1.0198
	B × 10^−3^	3.9815	2.7335	−0.2323
	*R* ^2^	0.9960	0.9930	0.9890
	RMSE	0.0163	0.0225	0.0305
	SSE	0.0074	0.0081	0.0121
	*X* ^2^	0.0003	0.0005	0.0009
Page	*k* × 10^−3^	8.0674	14.8538	2.3464
	n	0.8976	0.8568	1.3510
	*R* ^2^	0.9970	0.9970	0.9940
	RMSE	0.0143	0.0151	0.0206
	SSE	0.0057	0.0039	0.0064
	*X* ^2^	0.0002	0.0002	0.0005
Modified Page	*k* × 10^−3^	4.6539	7.3495	11.3124
	n	0.8976	0.8567	1.3510
	*R* ^2^	0.9970	0.9970	0.9940
	RMSE	0.0143	0.0151	0.0206
	SSE	0.0057	0.0039	0.0064
	*X* ^2^	0.0002	0.0002	0.0005
Henderson and Pabis	*k* × 10^−3^	4.3376	6.7572	11.9808
	a	0.9613	0.9663	1.0197
	*R* ^2^	0.9950	0.9930	0.9890
	RMSE	0.0162	0.0220	0.0267
	SSE	0.0076	0.0082	0.0121
	*X* ^2^	0.0003	0.0005	0.0008
Midilli–Küçük	*k* × 10^−3^	7.5998	16.6187	2.2864
	n	0.9056	0.8320	1.3585
	a	0.9876	0.9969	1.0042
	B × 10^−5^	−0.1262	−1.2743	0.7306
	*R* ^2^	0.9970	0.9980	0.9950
	RMSE	0.0143	0.0144	0.0216
	SSE	0.0055	0.0031	0.0061
	*X* ^2^	0.0002	0.0002	0.0005

**Table 2:** Experimental responses obtained from the central composite design (CCD).

Temperature (°C)	Particle Size (mm)	Thymol-Equivalent Response (ppm; Original Extract)	TPC (mg GAE g^−1^)	Yield (%)	Thymol-Equivalent Content (mg TE g^−1^ Dry Leaves)
50	1.25	468.4 ± 50.1	141.2 ± 5.8	4.4 ± 0.5	16.7 ± 1.8
50	1.25	435.1 ± 66.11	143.0 ± 2.2	4.6 ± 0.3	15.5 ± 2.4
50	1.25	475.0 ± 64.1	145 ± 2.2	4.5 ± 0.4	17.0 ± 2.3
50	1.25	448.4 ± 58.3	134.8 ± 0.6	4.2 ± 0.7	16.0 ± 2.1
40	0.50	672.9 ± 86.9	165.8 ± 9.9	7.3 ± 0.9	24.0 ± 3.1
64	1.25	322.0 ± 48.9	74.2 ± 0.2	2.5 ± 0.5	11.5 ± 1.7
50	1.25	461.7 ± 62.1	133.4 ± 8.5	4.0 ± 1.8	16.5 ± 2.2
60	2.00	244.6 ± 37.2	62.8 ± 2.6	2.0 ± 0.3	8.7 ± 1.3
60	0.50	408.5 ± 47.2	124.4 ± 0.4	3.6 ± 0.5	14.6 ± 1.7
50	2.31	376.0 ± 35.7	149.3 ± 3.1	4.2 ± 0.2	13.4 ± 1.3
50	0.19	717.3 ± 97.4	193.4 ± 0.1	9.0 ± 1.4	25.6 ± 3.5
36	1.25	387.0 ± 58.8	101.5 ± 10.9	2.6 ± 0.2	13.8 ± 2.1
40	2.00	415.2 ± 82.9	149.2 ± 15.8	4.5 ± 0.6	14.8 ± 3.0

**Table 3:** Estimation of the relative error between the experimental and model-predicted values under the model-predicted conditions.

Response	Objective	Importance	Weight	Predicted	Experimental	Absolute Relative Prediction Error (%)
Thymol-equivalent response (ppm)	maximize	3	5	785.3	964.3 ± 6.7	18.6
TPC (mg GAE g^−1^)	maximize	3	3	206.4	195.4 ± 5.3	5.6
Yield (%)	maximize	3	1	10.1	15.42 ± 1.54	34.4

## Data Availability

The original contributions presented in this study are included in the article and Supplementary Material. Further inquiries can be directed to the corresponding author.

## Supplementary Materials

The following supporting information can be downloaded at https://www.mdpi.com/article/10.3390/foods15213795/s1, Table S1. Mathematical models applied to the drying curve of the study; Table S2. Values of the independent variables and their corresponding levels in the central composite design (CCD); Table S3. Diffusivity coefficient (*D_eff_*) during the drying *of Plectranthus amboinicus*; Table S4. Analysis of variance and regression coefficients for the cubic model fitted to the thymol-equivalent response (ppm); Table S5. Analysis of variance and regression coefficients for the cubic model fitted to total phenolic content (mg GAE g^−1^); Table S6. Analysis of variance and regression coefficients for the cubic model fitted to extraction yield (%); Figure S1. Calibration curves for thymol and carvacrol determined by UV–Vis spectrophotometry.
