# Supplementary material for: Effect of Drying Preconditioning and Particle Size on Extractable Constituents from Plectranthus amboinicus Leaves by Ultrasound-Assisted Extraction
Source: Foods. 2026 Oct 23;15(21):3795. doi: 10.3390/foods15213795 (PMC13762723; doi:10.3390/foods15213795)
Supplement: Supplementary file 1 [file foods-15-03795-s001.zip › foods-4616079-supplementary.pdf]

foods

Article

Effect of Drying Preconditioning and Particle Size on Extractable Constituents from *Plectranthus amboinicus* Leaves by Ultrasound-Assisted Extraction

Dayana Portillo-Izquierdo[1], Diego C. Durán[1], Oswaldo Osorio-Mora[2], Ramiro Torres-Gallo[3] and Diego F. Tirado[1,*]

[1] Biological Engineering Program, Dirección Académica, Universidad Nacional de Colombia, Sede de La Paz, La Paz 202017, Colombia; dportilloi@unal.edu.co (D.P.-I.); diduran@unal.edu.co (D.C.D.); dtiradoa@unal.edu.co (D.F.T.)
[2] Facultad de Ingeniería Agroindustrial, Grupo de investigación GAIDA, Universidad de Nariño, Pasto 520002, Colombia; osorio_oswaldo@udenar.edu.co
[3] Department of Agroindustrial Engineering, Facultad de Ingeniería, Universidad del Atlántico, Puerto Colombia 081001, Colombia; ramirotorres@mail.uniatlantico.edu.co
* Correspondence: dtiradoa@unal.edu.co

Table S1. Mathematical models applied to the drying curve of the study.

Model name	Model	Reference
Page	$MR = exp(-kt^n)$	[24]
Modified page	$MR = \exp[-(kt)^n]$	[25]
Henderson and Pabis	$MR = a \cdot exp(-kt)$	[26]
Logarithmic	$MR = a \cdot exp(-kt) + b$	[27]
Midilli-Kuck	$MR = a \cdot exp(-kt^n) + bt$	[28]

MR: moisture ratio; t: drying time (min); *k, n, a,* and *b:* empirical parameters of the models.

Table S2. Values of the independent variables and their corresponding levels in the central composite design (CCD).

Independent variables	Code	Variable levels				
		-α/-1.414	-1	0	+1	α/+1.414
Temperature (°C)	X_1	36	40	50	60	64.
Particle size (mm)	X_2	0.19	0.50	1.25	2.00	2.31

Table S3. Diffusivity coefficient (D_{eff}) during the drying *of Plectranthus amboinicus*.

Drying temperature (°C)	D_{eff} (m^2s^{-1})	R^2
40	6.68×10^{-12}	0.992
50	1.35×10^{-11}	0.992
60	1.72×10^{-11}	0.993

Table S4. Analysis of variance and regression coefficients for the cubic model fitted to thymol-equivalent response (ppm).

Source	Sum of Squares	df	Coefficient	*p*-value
Model	1.914×10^5	6	–	< 0.0001 *
A-Temperature	2113.15	1	-22.98	0.0438 *
B-Particle size	1.022×10^5	1	-113.03	< 0.0001 *
AB	2200.08	1	23.45	0.0409 *
A^2	21371.21	1	-55.43	0.0002 *
B^2	11497.02	1	40.65	0.0010 *
AB^2	14719.16	1	-85.79	0.0005 *
Lack of Fit	932.16	2		0.2748 ns
Residual	1959.13	6		
Pure Error	1026.97	4		
Cor. Total	1.934×10^5	12		

Intercept = 457.71. R^2 = 0.9899; Adjusted R^2 = 0.9797; Predicted R^2 = 0.9018; C.V. (%) = 4.03; Adeq Precision = 34.26.

* Significant at $p \leq 0.05$; ns: not significant.

Table S5. Analysis of variance and regression coefficients for the cubic model fitted to total phenolic content (mg GAE g^{-1}).

Source	Sum of Squares	df	Coefficient	*p*-value
Model	14861.78	6	–	< 0.0001 *
A-Temperature	372.64	1	-9.75	0.0112 *
B-Particle size	2470.05	1	-17.58	< 0.0001 *
AB	506.25	1	-11.25	0.0056 *
A^2	5050.87	1	-27.35	< 0.0001 *
B^2	1560.02	1	14.98	0.0003 *
AB^2	975.72	1	-22.20	0.0011 *
Lack of Fit	55.97	2		0.4531 ns
Residual	171.24	6		
Pure Error	115.27	4		
Cor. Total	15033.02	12		

Intercept = 139.68. R^2 = 0.9886; Adjusted R^2 = 0.9772; Predicted R^2 = 0.9221; C.V. (%) = 4.04; Adeq Precision = 32.63

* Significant at $p \leq 05$; ns: not significant.

Table S6. Analysis of variance and regression coefficients for the cubic model fitted to extraction yield (%).

Source	Sum of Squares	df	Coefficient	*p*-value
Model	43.29	7	–	< 0.0001 *
A-Temperature	0.0040	1	-0.0318	0.8034
B-Particle size	11.76	1	-1.71	< 0.0001 *
AB	0.3422	1	0.2925	0.0606
A^2	6.54	1	-0.9694	0.0001 *
B^2	7.90	1	1.07	< 0.0001 *
A^2B	0.7254	1	0.6022	0.0170 *
AB^2	4.62	1	-1.52	0.0003 *
Lack of Fit	0.0861	1		0.2677 ns
Residual	0.2939	5		
Pure Error	0.2078	4		
Cor. Total	43.59	12		

Intercept = 4.34. R^2 = 0.9933; Adjusted R^2 = 0.9838; Predicted R^2 = 0.8661; C.V. (%) = 5.51; Adeq Precision = 35.92

* Significant at $p \leq 0.05$; ns: not significant.

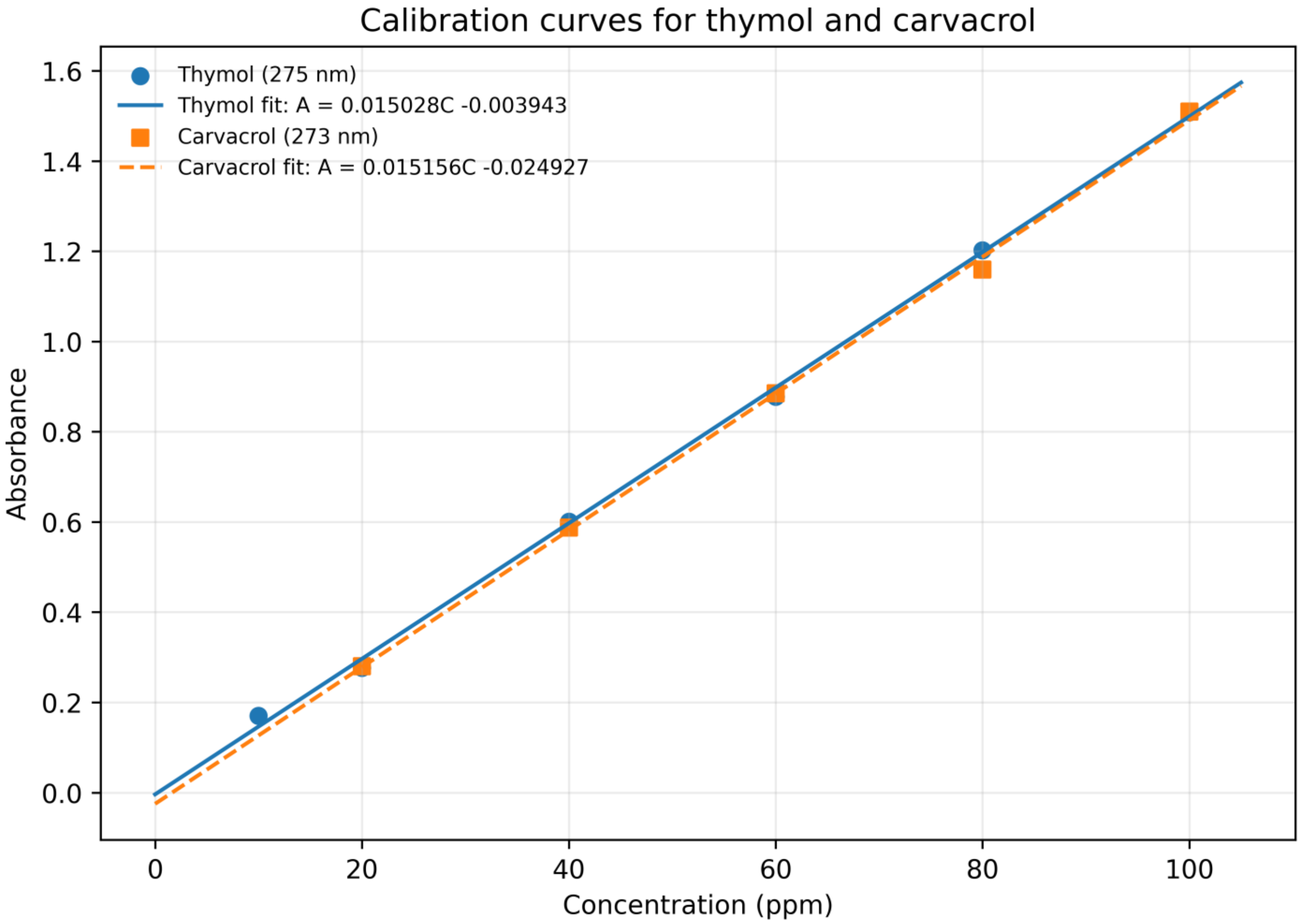

Figure S1. Calibration curves for thymol and carvacrol determined by UV–Vis spectrophotometry.
